# Perovskite LaFeO_3_/montmorillonite nanocomposites: synthesis, interface characteristics and enhanced photocatalytic activity

**DOI:** 10.1038/srep19723

**Published:** 2016-01-18

**Authors:** Kang Peng, Liangjie Fu, Huaming Yang, Jing Ouyang

**Affiliations:** 1 Centre for Mineral Materials, School of Minerals Processing and Bioengineering, Central South University, Changsha 410083, China; 2 Key Laboratory for Mineral Materials and Application of Hunan Province, Central South University, Changsha 410083, China; 3 State Key Laboratory of Powder Metallurgy, Central South University, Changsha 410083, China

## Abstract

Perovskite LaFeO_3_/montmorillonite nanocomposites (LaFeO_3_/MMT) have been successfully prepared via assembling LaFeO_3_ nanoparticles on the surface of montmorillonite with citric acid assisted sol-gel method. The results indicated that the uniform LaFeO_3_ nanoparticles were densely deposited onto the surface of montmorillonite, mainly ranging in diameter from 10 nm to 15 nm. The photocatalytic activity of LaFeO_3_/MMT was evaluated by the degradation of Rhodamine B (RhB) under visible light irradiation, indicating that LaFeO_3_/MMT exhibited remarkable adsorption efficiency and excellent photocatalytic activity with the overall removal rate of RhB up to 99.34% after visible light irradiation lasting for 90 min. The interface characteristic and possible degradation mechanism were explored. The interface characterization of LaFeO_3_/MMT suggested that LaFeO_3_ nanoparticles could be immobilized on the surface of montmorillonite with the Si-O-Fe bonds. The abundant hydroxyl groups of montmorillonite, semiconductor photocatalysis of LaFeO_3_ and Fenton-like reaction could enhance the photocatalytic degradation through a synergistic effect. Therefore, the LaFeO_3_/MMT is a very promising photocatalyst in future industrial application to treat effectively wastewater of dyes.

Nanoscale composite materials via supporting nanoparticles onto another matrix exhibit novel structure and distinguished performance in catalysis^1^,^2^,^3^,^4^,^5^, optics^6^,^7^, electronics^8^,^9^, and other applications^10^. The methods developed for the synthesis of nanoparticles onto the matrix mainly include precipitation^11^,^12^, sol-gel^13^,^14^, microwave-assisted synthesis^15^,^16^, phase transitions and hydrothermal process^17^. The matrix can disperse the nanoparticles and synergize properties, and the interface structure between phases can elicit new functional features. In recent years, the Nanocomposites with semiconductor nanoparticles assembled on substrates have received much attention for photocatalysis application, which has a very promising prospect in future industrial application of wastewater treatment^18^,^19^.

Lanthanum ferrite (LaFeO_3_) is an important P-type semiconductor with narrow band gaps of 2.65 eV and one of the most common perovskite type oxide. Perovskite type oxides have a general formula ABO_3_, where position A is occupied by the rare earth ion, and position B by the transition metal ion. LaFeO_3_ possesses significant physical and chemical properties, and has been applied in advanced technologies such as electronic and magnetic materials^20^, solid oxide fuel cells^21^, gas sensor^22^ and catalysts^23^,^24^. The properties of LaFeO_3_ nanoparticles depend on structure, morphology, and size, which are strongly influenced by the synthesis process. Several methods have been reported for the synthesis of perovskite LaFeO_3_ nanoparticles, such as sol-gel method, co-precipitation, solution combustion^25^, microemulsion^26^, hydrothermal process, thermal decomposition^27^. Among these, the sol-gel method is considered to be one of the most successful methods for synthesizing uniform and small LaFeO_3_ nanoparticles in recent decades. However, the LaFeO_3_ nanoparticles are easily agglomerated for high surface energy, which leads to serious decrease of the performance. One of effective solutions is dispersing the nanoparticles on the matrices, and the matrices provide heterojunctions for electrons and holes that restricted the charge recombination. Up to date, various matrices have been utilized for supporting catalyst nanoparticles, such as mesoporous materials^28^,^29^,^30^,^31^,^32^,^33^, cordierite monolith^34^, TiO_2_ films^35^, TiO_2_ nanotube^36^,^37^,^38^,^39^,^40^, ZrO_2_^41^, and clay minerals^42^,^43^. Among these matrices, the support materials with two dimension (2D) morphology have excellent performance enhancements for high specific surface area and favorable dispersion effect.

Montmorillonite is one of the most abundant clay mineral with natural 2D sheet-like morphology. The montmorillonite sheet consists of three layer structural units (TOTs), which is composed of a central AlO_4_(OH)_2_ octahedral sheet and two SiO_4_ tetrahedral sheets, and it possesses many hydroxyl groups. These hydroxyl groups and natural sheet-like morphology make montmorillonite become a promising matrix material for assembling LaFeO_3_ nanoparticles. Montmorillonite has been successfully used to remove dyes from aqueous solutions^44^ for its excellent adsorption capability and large specific surface area. Considering LaFeO_3_ nanoparticles possess great catalytic performance in dye water treatment, it could be possible for montmorillonite supported LaFeO_3_ nanoparticles to be used in organic dyes degradation in wastewater by the synergistic effect of adsorption and catalysis.

More than 0.7 million tons of synthetic organic dyes are produced annually worldwide^45^. Rhodamine B is one of cationic xanthene dyes used in printing, textile, photographic industries and fluorescent material. It not only irritates the skin, eyes and respiratory tract, but might cause carcinogenicity, reproductive and neural toxicity towards humans and animals. It is increasingly important to treat the highly colored wastewater with hazardous industrial chemical dyes efficiently. So far, the promising approaches to remove dyes from aqueous solutions mainly include adsorption engineering and photocatalysis technology^46^,^47^.

In this study, we first demonstrated sol-gel synthesis of perovskite LaFeO_3_/MMT via assembling LaFeO_3_ nanoparticles on the surface of montmorillonite sheets. A series of LaMO_3_/MMT (M=Fe,Co,Ni) samples were synthesized in the same way for screening tests. Moreover, the photocatalytic activities of LaMO_3_/MMT were evaluated in detail by the photodegradation of RhB under visible light irradiation. The possible degradation mechanism was investigated, and the interface structure feature of LaMO_3_/MMT was characterized.

## Results

**Figure 1:**
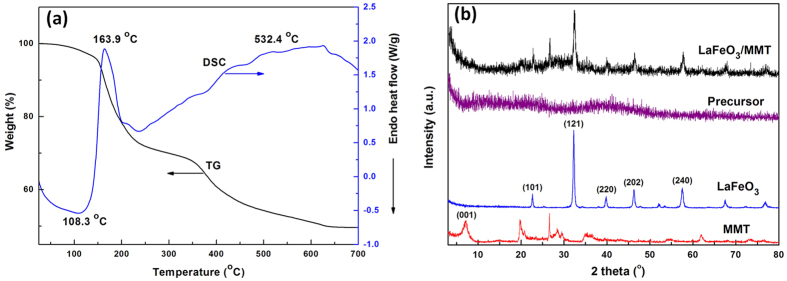
Formation and phase structure of the samples. (**a**) TG-DSC curves of LaFeO_3_/MMT precursor and (**b**) XRD patterns of MMT, LaFeO_3_, precursor and LaFeO_3_/MMT.

The TG-DSC curves of the precursor of LaFeO_3_/MMT are shown in Fig. 1a. The sample showed a slight weight loss of about 1.8% up to 108.3 °C with endothermic phenomenon, which was ascribed to the loss the physically absorbed water in the gel. A distinct exothermic peak accompanying massive weight loss of approximately 30% could be observed at 163.9 °C, which indicated the thermal transformation of citric acid. The exothermic process between 300 to 500 °C was attributed to the combustion decomposition of citric acid, and it led to a weight loss of 12.6%. With the temperature increasing, a slight loss process high than 500 °C was mainly ascribed to desorption of residual hydroxyl group. A slight exothermic peak at about 532.4 °C was caused by the gradual crystallization of LaFeO_3_. Therefore, 600 °C was chosen as the calcination temperature for preparing LaFeO_3_/MMT.

The phase and crystallinity of the samples were determined using powder XRD measurement (Fig. 1b). The XRD pattern of MMT clearly displayed a typical reflection at 2*θ* = 7.26° (001) for Na-montmorillonite, indicating the d_001_ spacing of 1.217 nm. The diffraction peaks at 19.76°, 20.89°, 35.08° and 61.96° were corresponding to the main mineral component of Na-montmorillonite. The diffraction peaks at 26.6° was ascribed to the impurity of quartz. As for the pure LaFeO_3_, all diffraction peaks were readily indexed to the perovskite phase with an orthorhombic structure. The lattice parameters of pure LaFeO_3_ were in excellent accordance with the standard powder diffraction data (JCPDS card No. 37–1493), and the average crystallite size was estimated to be 36.4 nm in diameter by the Scherrer’s equation. There was no peak of LaFeO_3_ phase in the XRD pattern of LaFeO_3_/MMT precursor, indicating the precursor was amorphous before calcined. The XRD pattern of LaFeO_3_/MMT presented the main diffraction peaks of montmorillonite and LaFeO_3_, and no new diffraction peaks occurred. The average crystallite size of LaFeO_3_ in LaFeO_3_/MMT was 15.5 nm, which was smaller than that of pure LaFeO_3_.

**Figure 2:**
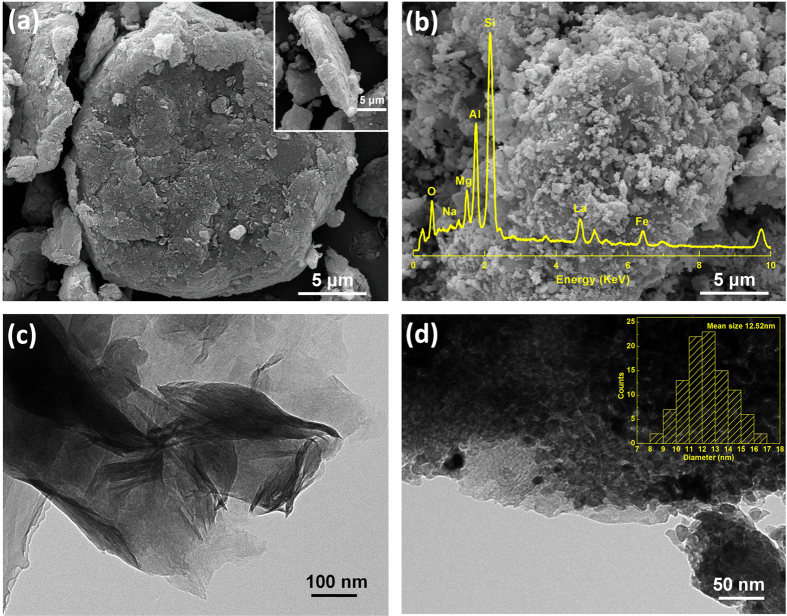
Morphologies of the samples. SEM images of (**a**) MMT, the inset shows the side view, and (**b**) LaFeO_3_/MMT, the inset shows the corresponding EDS spectrum; and TEM images of (**c**) MMT and (**d**) LaFeO_3_/MMT, the inset shows the corresponding size distribution diagram of LaFeO_3_ nanoparticles.

The general morphologies of the samples were investigated by SEM. The front and side images of MMT indicated that montmorillonite sheets with 15~20 μm in width and 0.5~1 μm in thickness possessed smooth surface without contamination (Fig. 2a). The LaFeO_3_ prepared by sol-gel method exhibited agglomeration of the particles with irregular shapes (Figure S1). From the SEM image and the EDS spectrum of LaFeO_3_/MMT (Fig. 2b), the LaFeO_3_ nanoparticles were successfully assembled on the surface of montmorillonite sheets. It was clear that the diameter of LaFeO_3_ particles on the montmorillonite sheets was less than that of pure LaFeO_3_ particles, which agreed well with the XRD results. Therefore, The LaFeO_3_ particles assembled on montmorillonite sheets could have larger surface area and more catalytic reaction sites. The EDS spectrum reveal that LaFeO_3_/MMT was mainly comprised of Si K (28.09 wt%), Al K (8.32 wt%), Mg K (1.52 wt%), Na K (1.48 wt%) and O K (19.82 wt%), as well as La K (29.19 wt%) and Fe K (11.57 wt%).

TEM characterization was adopted to obtain the further structural insights of MMT and LaFeO_3_/MMT. The samples for TEM had been dispersed fully in ethyl alcohol by sonicated for 30 min. It could be observed that the dispersed montmorillonite exhibited a 2D nanosheet with about 500 nm in width (Fig. 2c). The precursor of LaFeO_3_ could be absorbed on the surfaces of montmorillonite nanosheets, and the LaFeO_3_ crystals were generated from the precursor and assembled on the surfaces. Figure 2d showed that a large amount of LaFeO_3_ particles were well dispersed on the surfaces of montmorillonite. The diameter of LaFeO_3_ particles was mainly distributed around 10~15 nm, and the mean size was 12.52 nm. Therefore, it was expected that the growth of the LaFeO_3_ particles could be effectively controlled to a nano-size level via the cooperation of citric acid supported on montmorillonite.

**Figure 3:**
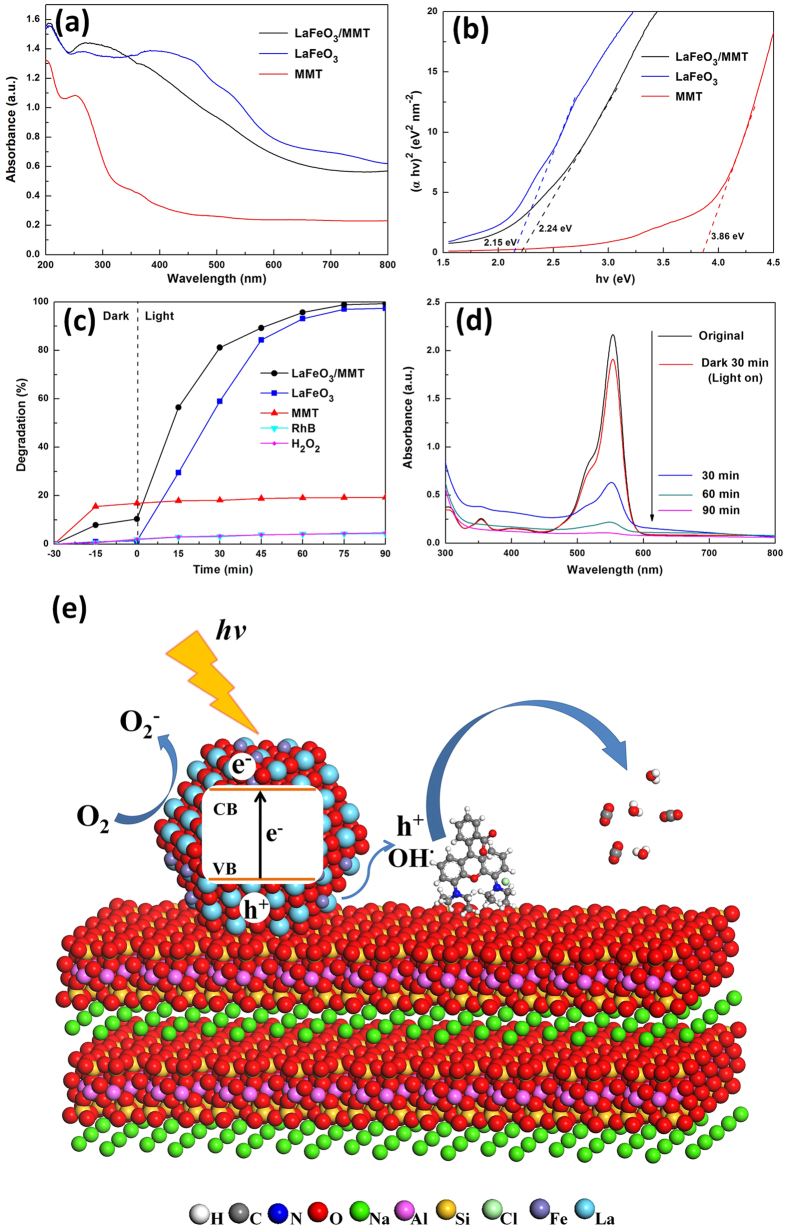
Photocatalytic activity and proposed reaction mechanism of the samples. (**a**) UV–vis diffuse reflectance spectra of MMT, LaFeO_3_ and LaFeO_3_/MMT, (**b**) the corresponding plots of (*αhv*)^2^ vs. photon energy (*hv*), (**c**) Photocatalytic degradation of RhB with H_2_O_2_, MMT, LaFeO_3_ and LaFeO_3_/MMT, (**d**) UV-vis absorption changes of RhB solution during the photocatalytic reaction with LaFeO_3_/MMT, and (**e**) schematic illustration of the degradation mechanism for the photocatalytic reaction route.

The energy band structure feature was considered as a key factor for photocatalytic activity. The samples were characterized by UV–vis diffuse reflectance spectroscopy to explore their optical properties. Figure 3a shows the UV–vis diffuse reflectance spectra of MMT, LaFeO_3_ and LaFeO_3_/MMT. The LaFeO_3_ and LaFeO_3_/MMT exhibited a broad absorption band in the wavelength ranging from 400 to 620 nm, which could be attributed to the electronic transition from the valance band to the conduction band. The LaFeO_3_ and LaFeO_3_/MMT particles could absorb considerable amounts of visible light, which implied their potential applications as visible light driven photocatalysts. Under visible light irradiation, the photocatalyst absorbed light above 400 nm, which contributed to the formation of photogenerated electron-hole pairs. The MMT exhibited a weak absorption of visible light.

The band gap energy (E_g_) could be estimated from a plot of (*αhv*)^2^ versus photon energy (*hv*), where *α, h* and *v* were absorption coefficient, Planck constant and light frequency, respectively. The intercept of the tangent to the *x*-axis could give a good approximation of the band gap energy. The band gap energy was estimated to be about 2.15 eV for LaFeO_3_ (Fig. 3b), 2.24 eV for LaFeO_3_/MMT and 3.86 eV for MMT. The values in this research were comparable with the previous results reported in literature^48^. The large band gap energy of LaFeO_3_/MMT compared with LaFeO_3_ could be attributed to the less size of LaFeO_3_ nanoparticles assembled on montmorillonite, which suggested that the band gap of LaFeO_3_/MMT was suitable for the photocatalytic decomposition of organic contaminants via activation by visible light irradiation.

The photocatalytic activity of LaFeO_3_/MMT was studied using the degradation of RhB as a probe reaction, and the results were compared with those of LaFeO_3_ and MMT (Fig. 3c). There was minimal change in the RhB solution without catalyst, indicating that the RhB solution could not self-decompose. RhB is hardly degraded with H_2_O_2_ only (Fig. 3c). Adsorption was pre-conducted in dark for 30 min, and then photocatalysis proceeded under visible light at *t* = 0. In the adsorption phase, adsorption equilibrium was quickly reached and only physical adsorption was observed for the different samples. The adsorption efficiencies of MMT, LaFeO_3_ and LaFeO_3_/MMT were 16.83%, 1.39% and 10.38% for RhB, respectively. The amount of RhB adsorption on the MMT was most, this phenomenon was mainly related to structure of montmorillonite. Montmorillonite was a 2:1-type clay mineral wherein each layer contained two SiO_4_ tetrahedral sheet and an AlO_4_(OH)_2_ octahedral sheet. The aluminum cations coordinated by hydroxyl groups was conducive to the adsorption of RhB. LaFeO_3_/MMT exhibited much higher adsorptive ability than LaFeO_3_, which was ascribed to the larger surface areas of LaFeO_3_/MMT.

With LaFeO_3_/MMT as the catalyst, the overall removal rate of RhB was up to 99.34% after visible light irradiation lasting for 90 min. For the case of LaFeO_3_, the removal rate of RhB was 97.33% via photocatalytic degradation. As for MMT, the concentration of RhB decreased by only 19.29%, which physic adsorption was dominant. However, at the beginning of 30 min under the visible light, the removal rate of RhB was 81.16% for LaFeO_3_/MMT, much higher than that for LaFeO_3_, which was only 58.96%. The LaFeO_3_/MMT had greater photocatalytic reaction rate than LaFeO_3_. Figure 3d shows the temporal evolution of the UV-vis absorption spectra of RhB solution during the photocatalytic reaction with LaFeO_3_/MMT. RhB solution shows a major absorption peak at 554 nm. Clearly, the intensity of the absorption peak gradually declined with increasing photocatalytic reaction time, which represented the concentration of RhB decreasing by degrees. There was no obvious absorption peak in the UV-vis absorption spectrum of the final degradation solution (Fig. 3d), indicating that RhB was degraded into H_2_O, CO_2_ and non-toxic products^49^. It is observed that the RhB solution was almost degraded completely by LaFeO_3_/MMT in 90 min.

The photocatalytic performance of LaFeO_3_/MMT was higher than that of LaFeO_3_, which could be attributed to the specific properties of montmorillonite. The high adsorption property of montmorillonite contributed to the synergistic removal of RhB. The abundant hydroxyl groups could trap light holes and generate active hydroxyl radicals^50^. Therefore, montmorillonite with a natural sheet-like morphology was an excellent host material for photocatalysis, which could enhance the property of LaFeO_3_ nanoparticles. The montmorillonite sheets could prevent LaFeO_3_ nanoparticles aggregation and control the LaFeO_3_ particle size effectively. The uniform and small LaFeO_3_ nanoparticles were obtained on the surface of montmorillonite, which contributed to enhance the LaFeO_3_ photocatalysis behavior.

The degradation mechanism for the photocatalytic reaction route of LaFeO_3_/MMT is schematically illustrated in Fig. 3e. Under the irradiation of visible light with energy greater than the threshold, the photogenerated electrons (e^−^) were transferred to the conduction band (CB) from the valence band (VB) leaving the positive holes (h^+^) in the VB. The electron–hole recombination was unavoidable; however, the abundant hydroxyl groups of LaFeO_3_/MMT could trap photoinduced holes to yield hydroxyl radicals (OH^·^) and decrease the recombination rate of electron-hole pairs. The conduction band electrons probably reacted with oxygen molecules (O_2_) to produce superoxide radical anions (O_2_^−^), which could yield hydroxyl radicals. These hydroxyl radicals and photoinduced holes were the strong oxidizing agents to degrade RhB. The high adsorption property of LaFeO_3_/MMT could form the higher apparent concentration of RhB around the surface of catalyst, which further enhanced the photocatalytic activity.

## Discussion

**Figure 4:**
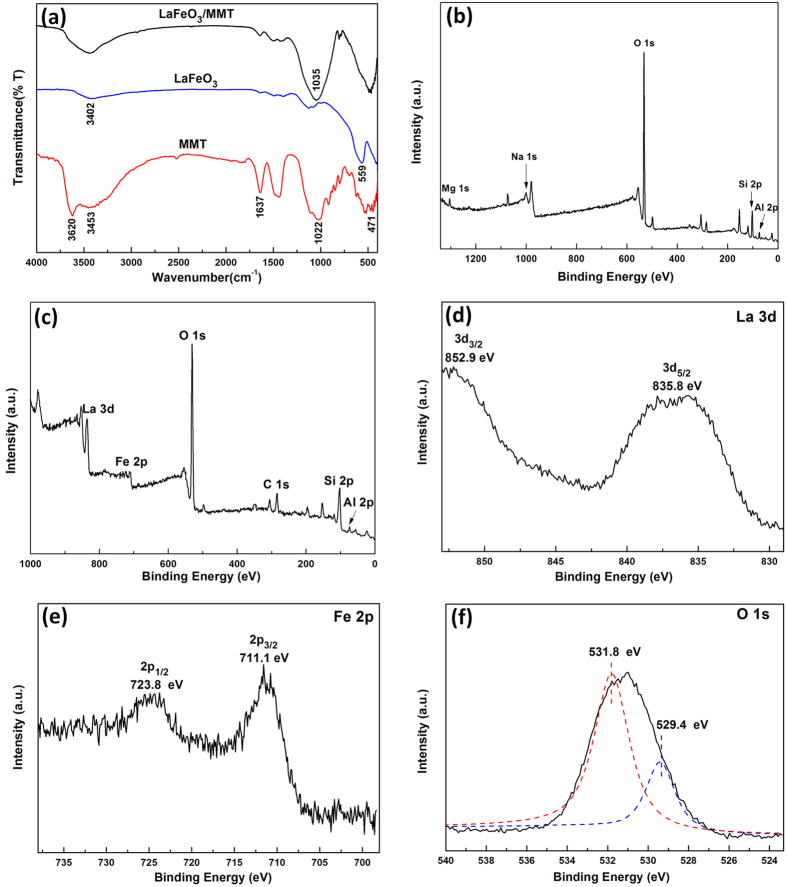
Interface characteristics of the samples. (**a**) FTIR spectra of MMT, LaFeO_3_ and LaFeO_3_/MMT, XPS survey spectrum of (**b**) MMT and (**c**) LaFeO_3_/MMT, and high-resolution scans of LaFeO_3_/MMT for (**d**) La 3d, (**e**) Fe 2p and (**f**) O 1s.

The interface characteristics were characterized by Fourier transform infrared spectra (FTIR) and X-ray photoelectron spectroscopy (XPS). The FTIR spectra clearly showed the vibrational bands of MMT, LaFeO_3_ and LaFeO_3_/MMT (Fig. 4a). For MMT, the bending vibration band of Si–O at 471 cm^−1^ and the stretching vibration band of O–Si–O at 1022 cm^−1^ indicated that the layered structure of montmorillonite consisted of silicon-oxygen tetrahedron. Bands close to 430, 471 and 518 cm^−1^ could be assigned to Si–O–Si, Si–O–Mg and Si–O–Al respectively. The obvious band at 3620 cm^−1^ was assigned to the –OH stretching region corresponding to the Al–O–H group. The broad band at 3453 cm^−1^ could be ascribed to the overlapping asymmetric and symmetric H–O–H stretching vibrations of H-bonded water, and OH bending vibration was observed at 1637 cm^−1^. Meanwhile, the Fe–O stretching vibration at 559 cm^−1^ appeared in LaFeO_3_, which was characteristic of the octahedral FeO_6_ group in the perovskite compounds. The band at 3402 cm^−1^ was attributed to the stretching vibration of O–H of absorbed water and hydroxyl group. The two bands at 1485 and1384 cm^−1^ were ascribed to the splitting of the asymmetric stretching of carbonates, indicating that La-carbonate species were formed on the surface of the LaFeO_3_ particles due to exposure to the ambient atmosphere^51^. In the FIIR spectrum of LaFeO_3_/MMT, the band at 3620 cm^−1^ assigned to the Al–O–H group disappeared. The stretching vibration band of O–Si–O shifted to 1035 cm^−1^ from 1022 cm^−1^, providing evidence that a chemical interaction existed between the LaFeO_3_ nanoparticles and montmorillonite.

The surface elemental composition and chemical status of MMT and LaFeO_3_/MMT were studied by XPS analysis. A wide survey scan of XPS spectra was taken, and the peaks corresponding to O, Na, Mg, Al and Si in spectrum of MMT (Fig. 4b) and the peaks corresponding to La, Fe, O, C, Si and Al in spectrum of LaFeO_3_/MMT (Fig. 4c) are observed. The La, Fe, O, Si and Al elements were derived from the surface of LaFeO_3_/MMT, while the carbon peak could be attributed to adventitious carbon on the surface of sample. The XPS La 3d and Fe 2p core-level spectra revealed that the lanthanum and iron atoms were in the formal chemical valance state of +3 in the LaFeO_3_/MMT^52^. The high-resolution spectrum of La 3d (Fig. 4d) showed two strong La peaks at 835.8 and 852.9 eV corresponding to spin-orbit splitting of 3d_5/2_ and 3d_3/2_ of La^3+^ ions in oxide form. The binding energies of Fe 2p_3/2_ and Fe2p_1/2_ were observed at 711.1 and 723.8 eV (Fig. 4e), which corresponded to the core level spectra of Fe^3+^ ions in their oxide form.

The high-resolution spectrum of O 1s (Fig. 4f) could be deconvolved into two peaks by Gaussian rule. The two peaks were wide and asymmetric, indicating that there were at least two kinds of O chemical states^49^. The major one corresponding to the binding energy at 531.8 eV was assigned to chemically absorbed hydroxyl oxygen species, while the shoulder one at 529.4 eV was attributed to the lattice oxygen species^53^. It could be concluded that there were abundant hydroxyl oxygen on catalyst, which was an important parameter to influence the catalytic performances in oxidation reaction. The high-resolution spectra of Si 2p and Al 2p for MMT and LaFeO_3_/MMT are shown in Figure S2. The Si 2p peak of LaFeO_3_/MMT had a slight shift compared with that of MMT, while the Al 2p peak had no obvious change. It indicated that LaFeO_3_ nanoparticles might be immobilized on montmorillonite with the Si–O–Fe bonds.

**Figure 5:**
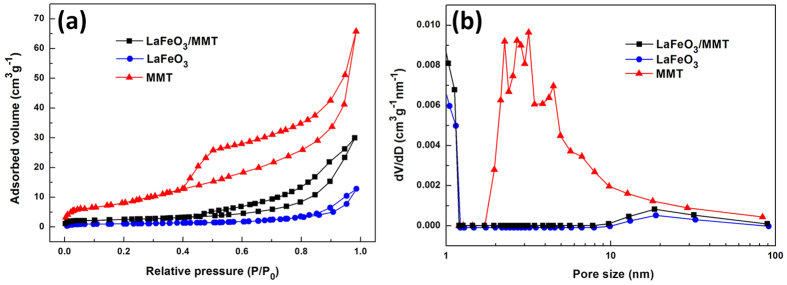
Porous textures of the samples. (**a**) Nitrogen adsorption-desorption isotherms and (**b**) BJH pore size distribution of MMT, LaFeO_3_ and LaFeO_3_/MMT.

The specific surface area and pore size distribution of MMT, LaFeO_3_ and LaFeO_3_/MMT were measured from nitrogen adsorption-desorption isotherms. The N_2_ adsorption-desorption isotherm curves of MMT and LaFeO_3_/MMT (Fig. 5a) exhibited type IV adsorption branch with a H3 hysteresis loop, which was characteristic of the mesoporous structure. However, LaFeO_3_ almost had no hysteresis loop, indicating it had a small surface area. The Brunauer–Emmett–Teller (BET) specific surface area of MMT, LaFeO_3_ and LaFeO_3_/MMT were 38.02, 5.37 and 13.15 m^2^g^–1^, respectively. The LaFeO_3_ powders had relatively small surface area due to the relatively large crystallite size and the agglomeration of the particles. Obviously, the LaFeO_3_/MMT sample had a higher specific surface area than LaFeO_3_, which could be attributed to the uniform distribution of LaFeO_3_ on MMT. The pore size distribution of samples could be calculated by Barrett-Joyner-Halenda (BJH) method (Fig. 5b). The MMT had mesopores with average pore size centralizing at 3 nm, while LaFeO_3_ exhibited a wide pore size distribution around 20 nm. The pore size distribution of LaFeO_3_/MMT was similar with that of LaFeO_3_, which might be because the LaFeO_3_ nanoparticles covered on the surface of MMT. The large surface area of LaFeO_3_/MMT compared with LaFeO_3_ could supply more adsorption and reactive sites, contributing to enhancing catalytic activity.

A series of LaMO_3_/MMT (M=Fe,Co,Ni) samples were synthesized via sol-gel method for screening tests. The XRD patterns of LaMO_3_/MMT showed that montmorillonite and perovskite phases existed in all LaMO_3_/MMT samples (Figure S3). The LaFeO_3_/MMT had the perovskite phase with an orthorhombic structure, while LaCoO_3_/MMT and LaNiO_3_/MMT had the perovskite phase with a rhombohedral structure. The crystallinity of LaNiO_3_/MMT was lower than that of LaFeO_3_/MMT and LaCoO_3_/MMT after calcined at 600 °C for 5 h. As for the La(FeCo)O_3_/MMT, La(FeNi)O_3_/MMT and La(CoNi)O_3_/MMT, the La(CoNi)O_3_/MMT possessed the highest crystallinity, which could be ascribed to the similar crystal structure of LaCoO_3_ and LaNiO_3_.

Figure S4a shows the UV–vis diffuse reflectance spectra of LaMO_3_/MMT (M=Fe,Co,Ni). The all LaMO_3_/MMT samples exhibited a broad absorption band in the wavelength ranging from 400 to 620 nm, and LaCoO_3_/MMT had higher absorption intensity for visible light than LaFeO_3_/MMT and LaNiO_3_/MMT. The band gap energy of LaFeO_3_/MMT was higher than that of LaCoO_3_/MMT and LaNiO_3_/MMT (Figure S4b). The band gap energies of La(FeCo)O_3_/MMT, La(FeNi)O_3_/MMT and La(CoNi)O_3_/MMT were between that of LaFeO_3_/MMT and LaCoO_3_/MMT, it might be because all LaMO_3_ phase possessed perovskite structure, and Fe, Co and Ni could replace with each other in crystal structure.

The photocatalytic activities of LaMO_3_/MMT (M=Fe,Co,Ni) were measured via the degradation of RhB, and the results are shown in Figure S5. All LaMO_3_/MMT samples exhibited good photocatalytic activity for RhB, and the LaFeO_3_/MMT possessed the highest removal rate up to 99.34%. Adsorption measurements for RhB showed that All LaMO_3_/MMT samples exhibited similar adsorption efficiencies ranging from 10% to 15%. LaFeO_3_/MMT presented better activity for RhB degradation than LaCoO_3_/MMT and LaNiO_3_/MMT. This could be attributed to the rapid decomposition of H_2_O_2_ on the surface of LaCoO_3_/MMT and LaNiO_3_/MMT^54^. The LaMO_3_/MMT samples with Fe exhibited better photocatalytic activity for RhB, which could be ascribed to the synergistic effect between the semiconductor photocatalysis and Fenton-like reaction^55^.

LaFeO_3_/MMT-0.5 and LaFeO_3_/MMT-2 were synthesized via sol-gel method to explore the optimized ratio of LaFeO_3_, and the photocatalytic activities of the samples were evaluated via the degradation of RhB (Figure S6). LaFeO_3_/MMT-0.5 exhibited relatively high adsorption efficiency but low photocatalytic activity. LaFeO_3_/MMT possessed the highest removal rate with appropriate adsorption efficiency and photocatalytic activity. Therefore, considering photocatalytic activity and material cost, LaFeO_3_/MMT was chosen as the final optimized catalyst, the weight percentage of LaFeO_3_ and MMT is 36.37% and 63.63% in the final LaFeO_3_/MMT catalyst, respectively.

In summary, perovskite LaFeO_3_/montmorillonite nanocomposites have been successfully synthesized via citric acid assisted sol-gel method. The uniform and small LaFeO_3_ nanoparticles were supported on the surface of montmorillonite, and the montmorillonite sheets could prevent LaFeO_3_ nanoparticles aggregation and control the LaFeO_3_ particle size effectively. The main chemical valences of La and Fe are +3 in the LaFeO_3_/MMT, and there are large amounts of hydroxyls on the surfaces of catalyst. With LaFeO_3_/MMT as the catalyst, the overall removal rate of RhB was up to 99.34% under visible light irradiation. The abundant hydroxyl groups could trap light holes and generate active hydroxyl radicals, and the high adsorption property of montmorillonite contributed to the synergistic removal of RhB. A series of perovskite LaMO_3_/MMT (M=Fe,Co,Ni) samples were synthesized via sol-gel method for photocatalytic degradation of RhB. The LaMO_3_/MMT samples with Fe exhibited better photocatalytic activity for the synergistic effect between the semiconductor photocatalysis and Fenton-like reaction. Therefore, the LaFeO_3_/MMT nanocomposites had prospective application to treat effectively wastewater of dyes.

## Methods

### Materials

Na-montmorillonite (MMT) was obtained from Zhejiang Sanding Technology Co. Ltd. (Zhejiang, China). It consisted primarily of montmorillonite (>97%) with minor impurity of quartz. The compositions of montmorillonite were determined by chemical analysis, which consisted of 61.53% SiO_2_, 19.25% Al_2_O_3_, 3.48% MgO, 1.41% Fe_2_O_3_, 2.83% Na_2_O, 2.52% CaO, 0.55% K_2_O, and 8.43% loss on ignition. La(NO_3_)_3_·6H_2_O, Fe(NO_3_)_3_·9H_2_O, Co(NO_3_)_3_·6H_2_O, Ni(NO_3_)_3_·6H_2_O, citric acid (C_6_H_8_O_7_·H_2_O) were provided by Sinopharm Chemical Reagent Co. Ltd. (Beijing, China). All reagents were analytical grade and used without further purification.

### Preparation

In a typical synthesis, La(NO_3_)_3_·6H_2_O (0.005 mol), Fe(NO_3_)_3_·9H_2_O (0.005 mol), citric acid (0.010 mol) were dissolved in 30 mL mix-solvent (H_2_O/EtOH 1:2) to yield a homogeneous solution. The solution was stirred for 10 min and sonicated for 10 min at room temperature. 2.000 g of montmorillonite was added and the mixture suspension was stirred and heated to 70 °C until it became gel status. The precursor was obtained after the gel dried at 90 °C for 24 h. The precursor was calcined at 600 °C for 5 h with a heating rate of 2 °C/min, and the final product was marked as LaFeO_3_/MMT. Other samples were also prepared in the same way, and the material formulas were listed in Table S1. For comparison, Pure LaFeO_3_ sample was prepared in the same way without montmorillonite.

### Characterization

Differential scanning calorimetry (DSC) and thermogravimetry (TG) analysis was conducted at a heating rate of 10 °C/min in air atmosphere using a NETZSCH STA449C thermal analyzer. The X-ray diffraction (XRD) patterns of the samples were recorded on a DX-2700 X-ray diffractometer with Cu K*α* radiation (*λ* = 0.15406 nm) at a scan rate of 0.02 °/s and at 40 kV and 40 mA. The morphology of the samples was observed using a JEOL JSM-6360LV scanning electron microscope (SEM) at an accelerating voltage of 5 kV, which equipped with EDS. Transmission electron microscope (TEM) images were obtained on a JEOL JEM-2100F microscope operating at 200 kV. The UV-vis diffuse reflectance spectra (UV-vis DRS) were obtained using a Shimadzu UV2450 UV-vis spectroscopy with an integrating sphere. Fourier transform infrared spectra (FTIR) of the samples were obtained between 4000 cm^−1^ and 400 cm^−1^ on a Nicolet Nexus 670 FTIR spectrophotometer using KBr pellets. X-ray photoelectron spectroscopy (XPS) measurements were performed on an ESCALAB 250 spectrometer. Nitrogen gas adsorption-desorption isotherms were obtained at 77 K using an ASAP 2020 surface area analyzer.

### Photocatalytic activity evaluation

Decolorization of Rhodamine B (RhB) in aqueous solution was selected as a probe reaction to evaluate the photocatalytic activity of LaFeO_3_/MMT. A 150 W high pressure mercury lamp was used as a light source with wave length *λ* > 400 nm. In a typical photocatalytic experiment, 100 mg of catalyst was dispersed in 100 mL RhB aqueous solution (0.02 mmol/L). Before visible light irradiation, the suspensions were magnetically stirred in the dark for 30 min to ensure the establishment of an adsorption-desorption equilibrium among the catalyst, RhB and water. 1 mL of 3% H_2_O_2_ was added into every vessel as oxidant to initiate the reaction. About 3 mL of analytical sample was drawn from the reaction suspension every 15 min, and the catalyst was removed by centrifugation. The reaction progress was monitored by measuring the absorbance (*A*) of the clarified solution at 554 nm using the UV-vis spectrophotometer. The degradation (%) was calculated from the formula: degradation (%) = (*A*_*0*_ − *A*)/*A*_*0*_ × 100%, where *A*_*0*_ was the initial absorbance, and *A* was the absorbance at homologous times.

## Additional Information

**How to cite this article**: Peng, K. *et al*. Perovskite LaFeO_3_/montmorillonite nanocomposites: synthesis, interface characteristics and enhanced photocatalytic activity. *Sci. Rep.* **6**, 19723; doi: 10.1038/srep19723 (2016).

## Supplementary Material

Supplementary Information
