# Supplementary material for: Perovskite LaFeO3/montmorillonite nanocomposites: synthesis, interface characteristics and enhanced photocatalytic activity
Source: Sci Rep. 2016 Jan 18;6:19723. doi: 10.1038/srep19723 (PMC4726031; doi:10.1038/srep19723)
Supplement: Supplementary Information [file srep19723-s1.pdf]

Supplementary Information

Perovskite $LaFeO_3$/montmorillonite nanocomposites: synthesis, interface characteristics and enhanced photocatalytic activity

Kang Peng, Liangjie Fu, Huaming Yang*, Jing Ouyang*

Table S1 The material formulation for the samples.

Samples	$La(NO_3)_3·6H_2O$ (mol)	$Fe(NO_3)_3·9H_2O$ (mol)	$Co(NO_3)_3·6H_2O$ (mol)	$Ni(NO_3)_3·6H_2O$ (mol)	Citric acid (mol)	MMT (g)
$LaFeO_3$/MMT	0.005	0.005	0	0	0.010	2.000
$LaCoO_3$/MMT	0.005	0	0.005	0	0.010	2.000
$LaNiO_3$/MMT	0.005	0	0	0.005	0.010	2.000
$La(FeCo)O_3$/MMT	0.005	0.0025	0.0025	0	0.010	2.000
$La(FeNi)O_3$/MMT	0.005	0.0025	0	0.0025	0.010	2.000
$La(CoNi)O_3$/MMT	0.005	0	0.0025	0.0025	0.010	2.000
$LaFeO_3$/MMT-0.5	0.0025	0.0025	0	0	0.005	2.000
$LaFeO_3$/MMT-2	0.010	0.010	0	0	0.020	2.000
$LaFeO_3$	0.005	0.005	0	0	0.010	0

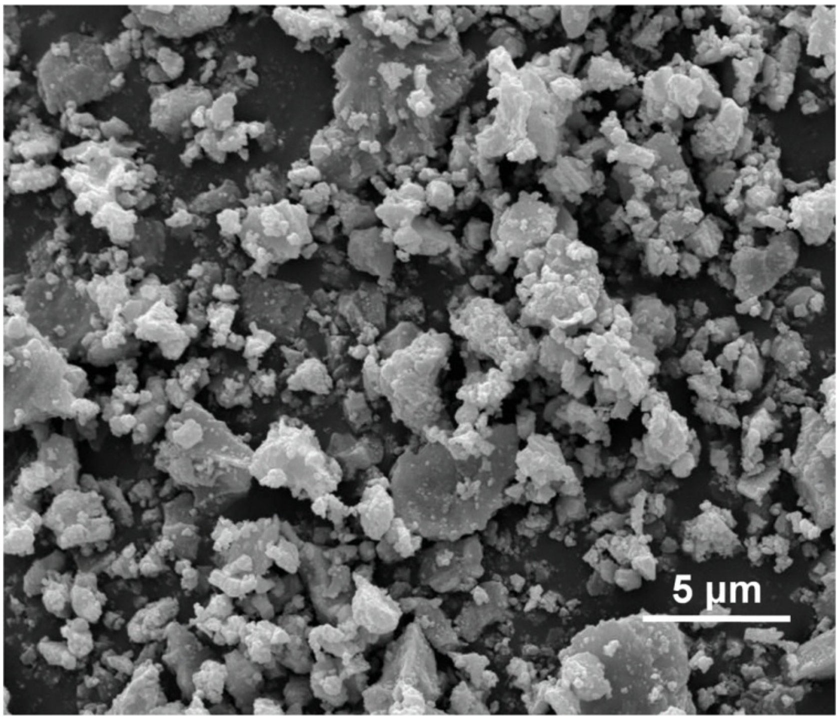

Figure S1 SEM image of $LaFeO_3$.

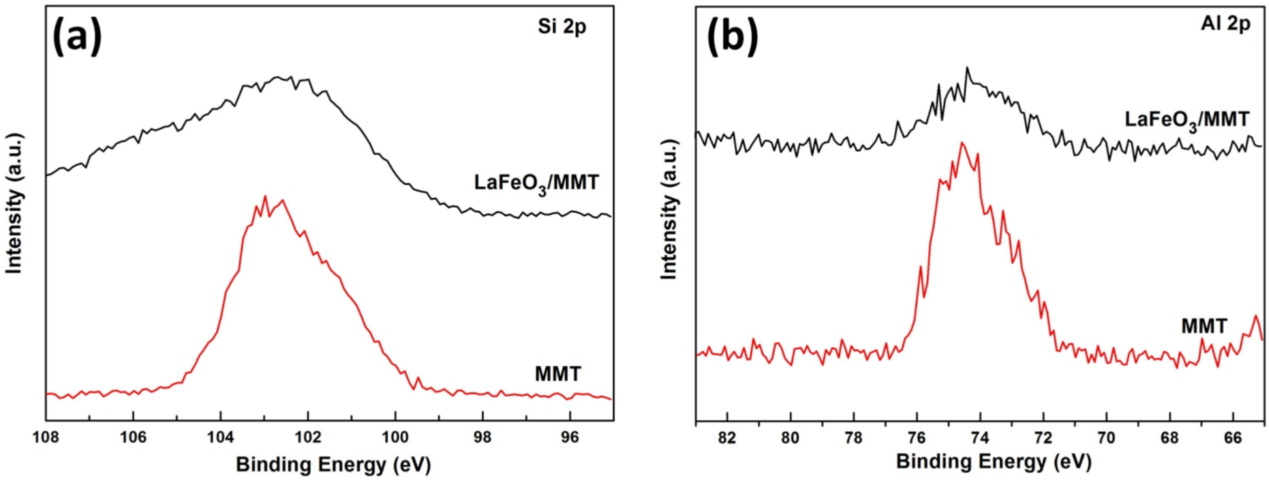

Figure S2 (a) Si 2p and (b) Al 2p XPS spectra of MMT and $LaFeO_3$/MMT.

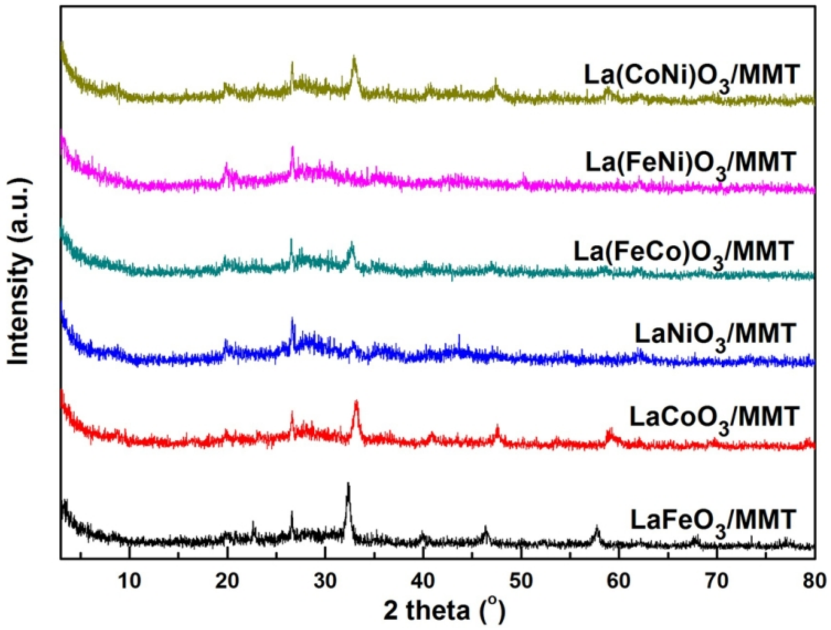

Figure S3 XRD patterns of $LaMO_3$/MMT (M=Fe,Co,Ni).

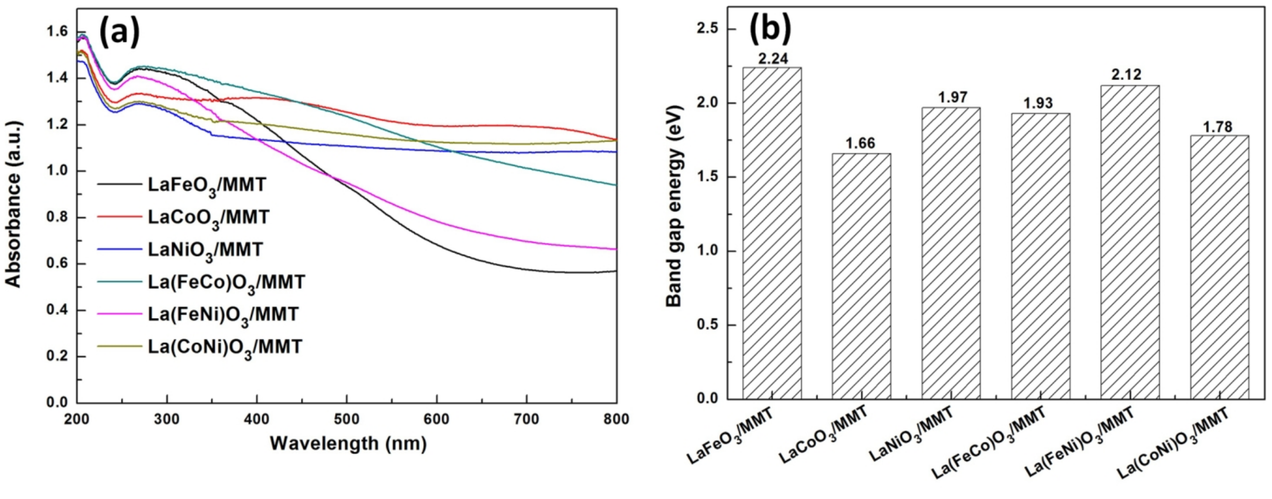

Figure S4 (a) UV–vis diffuse reflectance spectra and (b) band gap energies of $LaMO_3$/MMT (M=Fe,Co,Ni).

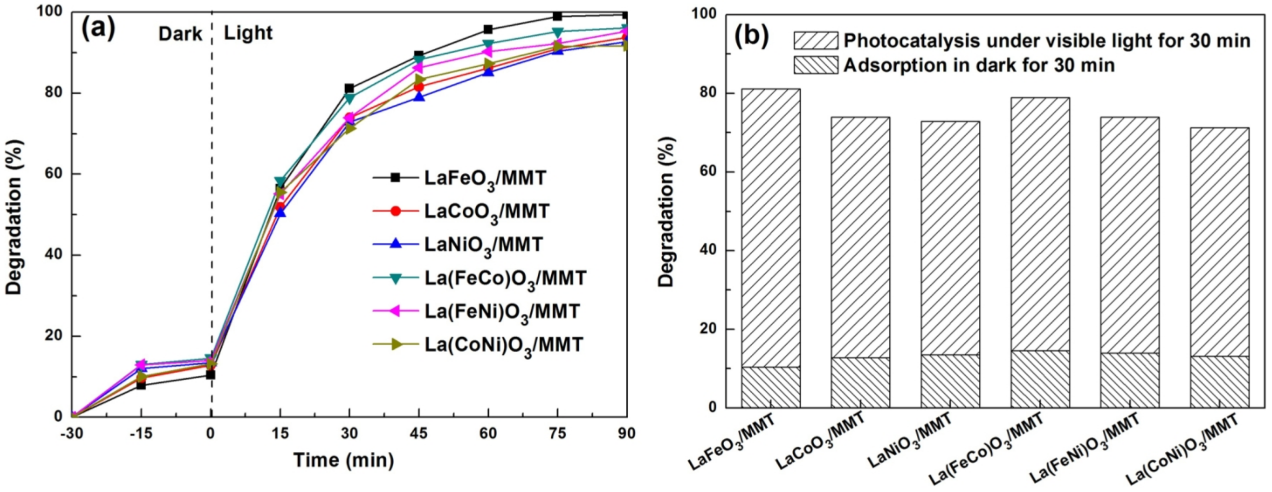

Figure S5 (a) Photocatalytic degradation of RhB with $LaMO_3$/MMT (M=Fe,Co,Ni), (b) Photocatalytic activities compare of $LaMO_3$/MMT.

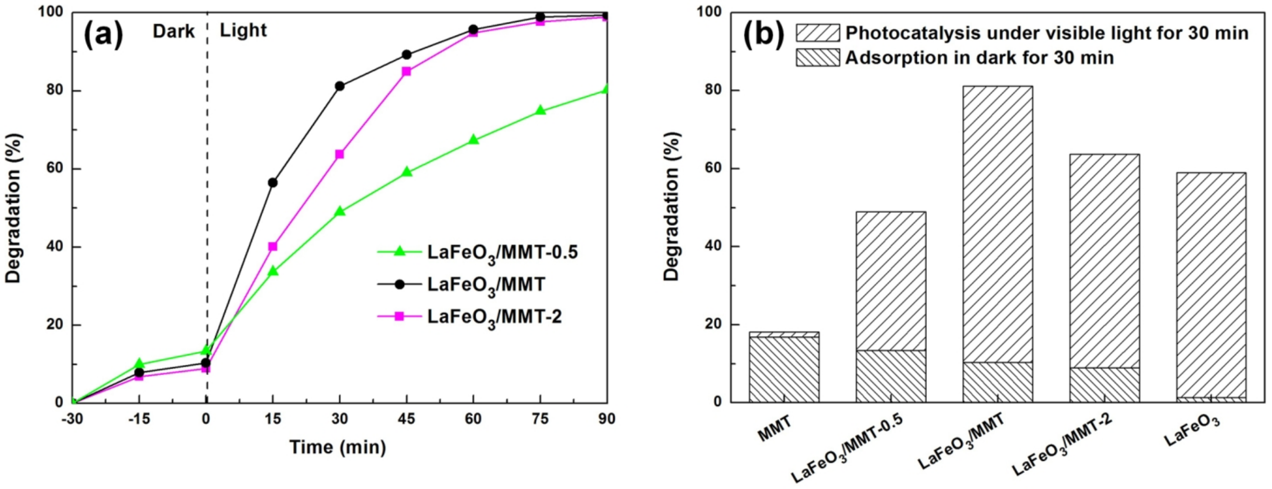

Figure S6 (a) Photocatalytic degradation of RhB with $LaFeO_3$/MMT-0.5, $LaFeO_3$/MMT and $LaFeO_3$/MMT-2, (b) Photocatalytic activities compare of MMT, $LaFeO_3$/MMT-0.5, $LaFeO_3$/MMT, $LaFeO_3$/MMT-2 and $LaFeO_3$
